# Clinical Validation and Excellent Interobserver Agreement of Volumetric Matching Micromotion Analysis (V3MA) in Total Knee Arthroplasty

**DOI:** 10.1002/jor.70049

**Published:** 2025-08-24

**Authors:** Nienke N. de Laat, Lennard A. Koster, Jessie E. Robertson, Rob G.H.H. Nelissen, Bart L. Kaptein

**Affiliations:** ^1^ Department of Orthopaedics Leiden University Medical Center Leiden The Netherlands; ^2^ Biomedical Engineering Program University of Manitoba Winnipeg Manitoba Canada

**Keywords:** arthroplasty, CT‐RSA, RSA, TKA, validation

## Abstract

CT‐based radiostereometric analysis (CT‐RSA) is an alternative to RSA to measure implant migration. We performed a clinical validation study using VoluMetric Matching Micromotion Analysis (V3MA) software for CT‐RSA. The aims of this study were to assess the agreement between V3MA and Model‐based RSA software (for RSA), and to determine the interobserver agreement in V3MA. On a subset of patients included in a clinical trial, knee prosthesis tibial implant migration was measured between 1 and 5 years postoperative with V3MA and Model‐based RSA software. V3MA and Model‐based RSA results were compared by assessing the mean differences and limits of agreement (mean ± 1.96* standard deviation) using Bland–Altman analysis. V3MA migration results of two observers were compared using intraclass correlation (ICC) and Bland–Altman analysis. Twenty‐four patients were included in the analysis. The mean difference (limits of agreement [LOA]) was −0.14 mm [−0.88 to 0.60] for maximum total point motion (MTPM). LOA for translations and rotations did not exceed ±0.5 mm and ±1°, respectively. The ICC (95% confidence interval) for MTPM between observers was 0.995 (0.989–0.998), and the mean difference [LOA] was 0.04 mm [−0.17 to 0.24]. We showed that between 1 and 5 years postoperative, V3MA migration results were comparable to those of Model‐based RSA for cemented tibial component migration in a clinical study. The interobserver variability showed excellent agreement for V3MA. Overall, V3MA is a valid alternative to Model‐based RSA for the analysis of tibial component migration in TKA with medium‐term follow‐up.

## 1 Introduction

Worldwide, more than 1 million total knee arthroplasties (TKA) are performed yearly [1] and numbers are increasing [2]. One of the most common reasons for failure of TKA is aseptic loosening (between 20% and 30%) [3]. Early and continuous implant migration is associated with late revision for loosening [4, 5, 6]. Migration analysis is used to evaluate new implant designs and surgical techniques and is considered a valuable tool in the orthopaedic research armamentarium [7, 8, 9, 10]. Mean tibial TKA migration for cemented arthroplasty, expressed as maximal total point motion (MTPM), greater than 1.1 mm at 6 months is considered unacceptable [11]. The current gold standard to measure implant migration is radiostereometric analysis (RSA). However, RSA requires bone markers, implant markers or digital implant models, special equipment, and trained radiology assistants. Alternatively, Computed‐tomography‐based RSA migration analysis (CT‐RSA) only requires standard clinical CT scans [12, 13, 14]. A CT‐RSA method, VoluMetric Matching Micromotion Analysis (V3MA), was validated for tibial components in TKA [15]. V3MA showed good accuracy and precision results in a phantom experiment, and showed promising clinical precision in a clinical proof of concept in 5 patients [15].

A recent systematic review showed that longer‐term clinical follow‐up studies and interobserver variability assessment of different CT‐RSA methods are needed to further validate CT‐RSA [16]. In the current paper, both of these issues are addressed. Our research questions were: (1) What is the agreement in 1 to 5‐year migration measurements of tibial components in TKA between CT‐RSA analysis using V3MA and model‐based RSA (mb‐RSA) using Model‐based RSA software; (2) What is the interobserver variability in CT‐RSA migration analysis of tibial TKA components using V3MA?

## 2 Materials and Methods

### 2.1 Patient Inclusion

In this prospective cohort study (level of evidence: level II), patients were included from a previous prospective randomized clinical trial (RCT) RSA study [17]. The study was conducted at the Orthopaedic department of Leiden University Medical Center (LUMC, Leiden) and compared migration of two cemented tibial implants (Persona PS and NexGen LPS, Zimmer Biomet) using mb‐RSA [17]. The original RCT protocol included RSA imaging at 1 and 5 years postoperative and an optional CT scan at 1 year postoperative. Fifty‐four patients out of 74 had a 1‐year CT scan and were invited for an additional CT scan 5 years after surgery. Only patients with a CT slice thickness < 1.0 mm in both CT scans were included in the study. Imaging for CT‐RSA and RSA was performed on the same day.

### 2.2 CT Scans

CT scans were acquired on a 320‐row detector CT scanner (Aquillion or Aquillion ONE, Canon Medical Systems). CT scans were conducted with a local clinical knee protocol. Images were reconstructed with a bone filter (convolution kernel FC30), and no metal artifact reduction algorithms were used. The matrix size was 512 × 512 in all images. CT‐scan details are provided in Supplemental Table 1.

### 2.3 Migration Measurement

**FIGURE 1:**
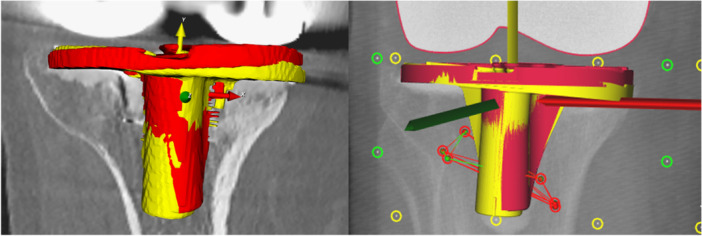
Visualization of the patient with the largest MTPM between 1 year (red) and 5 years (yellow) postoperative measured with CT‐RSA: 5.74 mm (Left), and visualization of the same patient with the largest MTPM between 1 year and 5 years postoperative measured with RSA: 5.07 mm (Right), The migration coordinate system is shown, with *X*‐axis (red, positive = medial translation), *Y*‐axis (yellow, positive = proximal translation) and *Z*‐axis (green, positive = anterior translation); MTPM = maximum total point motion; mm = millimeter. Note that the exact location and the orientation of the CT‐RSA and RSA coordinate systems vary slightly.

Migration of the tibial implant was measured with both CT‐RSA and mb‐RSA between 1 year (baseline) and 5 years (follow‐up) postoperative. These time points differ from regular RSA research, where migration is calculated with direct‐postoperative as the baseline [18]. The origin of the default coordinate systems of V3MA and Model‐based RSA is both in the center of gravity of the tibial implant model and is aligned with the coordinate system of the CT‐scanner and the calibration cage, respectively. Due to differences in these models (V3MA model is based on the mask obtained from segmentation, Model‐based RSA is a CAD‐based model), the coordinate systems are slightly different (Figure 1). Migration is presented for a right‐sided knee as maximum total point motion (MTPM in mm, main outcome parameter), translations (in mm), and rotations (in degrees) along three axes (*x*, *y*, and *z*), and their 3D vectors: Total Translation (TT) and Total Rotation (TR). Directions of migration are described according to the RSA coordinate system [18]. CT‐RSA analysis was conducted using V3MA (Version date 10/12/2024, RSA*core*, LUMC, Leiden, The Netherlands). This method involves defining masks for the bone and the implant in the baseline image, utilizing the underlying pixel intensities for registration of the bone and implant between baseline and follow‐up CT scans. This process enables the calculation of migration of the implant relative to the bone between baseline and follow‐up [15]. Mimics (Version 23.0, Materialise, Belgium) was used to define masks of the bone and implant. For each baseline image, thresholding and basic algorithms such as region growing, erosion, and dilation operations were used. The most proximal part of the tibia bone, a few millimeters under the tibial baseplate of the implant, was removed from the mask using cutting tools in Mimics; this region of the image was deteriorated by scattering caused by the tibial baseplate (Supplemental Figure 1). To avoid overlap between the bone and implant masks, the implant mask was subtracted from the bone mask. Mask creation and V3MA analysis were performed by two observers (N.L. and J.R.). Both observers used similar segmentation methods; however, certain steps, such as threshold definition and scatter removal, were determined at the observer's discretion. Bone length was defined as the length of the tibia bone distal to the tibial tray, which was imaged in both the baseline and follow‐up CT scan and therefore used for matching.

RSA was performed by an expert (L.K.) using Model‐based RSA software (v.4.2014, RSA*core*; LUMC, The Netherlands), adhering to the guidelines regarding marker matching [18]. RSA radiographs were acquired using our institutional standard RSA setup, including a uniplanar calibration cage with digital X‐ray equipment as described by Koster et al. [17].

### 2.4 Statistics

Bland–Altman plots were constructed to determine the mean difference and limits of agreement (LOA, mean ± 1.96 * standard deviation (SD)) between migration calculated with V3MA (N.L.) and Model‐based RSA, and between two observers independently operating V3MA (N.L. and J.R.) [19, 20]. The normal distribution of differences was checked visually by histograms and using Shapiro–Wilk [20]. The 95% confidence intervals (CI) were added to the Bland‐Altman plots to illustrate the magnitude of systematic differences [21]. If the 95% CI does not include 0, this indicates a bias between the two methods [21]. The interclass correlation coefficient was calculated with the 95% CI. Data analysis was conducted using R (version 3.6.1, R Foundation for Statistical Computing, Vienna, Austria) and Rstudio (version 1.1.456).

### Ethics

The included patients gave written informed consent for the initial RSA study, including CT acquisition 1 year postoperative, and the addendum to acquire CT images 5 years postoperative. The study protocol was approved by the institutional review board of LUMC under number: P13.277 and registered in ClinicalTrials.gov under NCT02269254 [17].

## 3 Results

Of the 54 patients that were invited for a 5‐year CT, 1 was revised, 2 had missing RSA data, 3 had a CT slice thickness ≥ 1 mm, 4 died, 10 did not give consent, and 10 were missing because of logistical reasons (partly as a result of covid). After exclusions, a total of 24 patients were included in this study.

**FIGURE 2:**
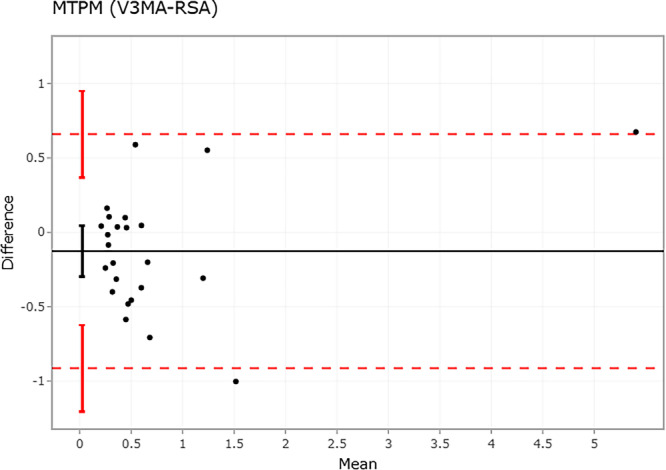
Bland–Altman plot of 24 patients (black dots) comparing V3MA (OBS 1) with Model‐based RSA on MTPM (in mm), showing the mean difference (black solid line) and the limits of agreement (red dashed lines). The vertical lines represent the 95% CI for the mean difference and limits of agreement. MTPM = maximum total point motion. mm = millimeter.

**FIGURE 3:**
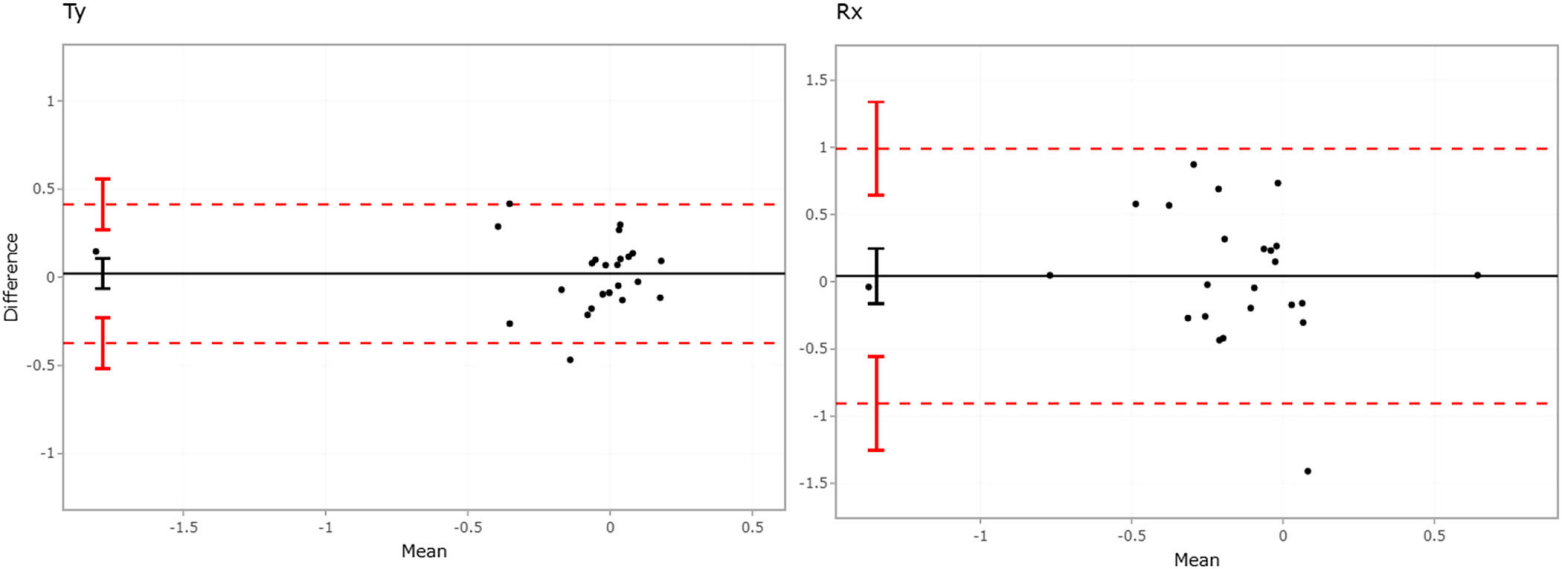
Bland–Altman plot of 24 patients (black dots) comparing V3MA (OBS 1) with Model‐based RSA on Subsidence (Ty (in mm)) and Tilting (Rx, in °), showing the mean difference (black solid line) and the limits of agreement (red dashed lines). The vertical lines represent the 95% CI for the mean difference and limits of agreement. mm = millimeter; ° = degree.

Mean (SD) tibial component MTPM between 1 and 5 years postoperative was 0.67 mm (1.13) and 0.80 mm (1.00) using CT‐RSA and mb‐RSA, respectively (Table 1). The mean difference (95% CI) in MTPM between CT‐RSA (observer 1) and mb‐RSA was −0.13 mm (±0.17), with limits of agreement [LOA] of −0.91 to 0.66 mm, indicating no significant bias (Figure 2). Mean differences (95% CI), [LOA] for subsidence and anterior tilting between CT‐RSA and mb‐RSA were 0.02 mm (±0.08), [−0.37 to 0.41] and 0.04° (±0.20), [−0.91 to 0.99] (Figure 3). The LOA for all translations and rotations remained within ±0.5 mm and ±1.0°, respectively (Supplemental Figure 2).

**TABLE 1:** Migration results of V3MA and Model‐based RSA.

	CT‐RSA OBS1 (*N* = 24)	CT‐RSA OBS2 (*N* = 24)	ICC CT‐RSA OBS1‐OBS2 (95% CI)	RSA (*N* = 24)
MTPM (mm)	0.67 (1.13)	0.64 (1.11)	0.995 (0.989 to 0.998)	0.80 (1.00)
TT (mm)	0.25 (0.37)	0.22 (0.35)	0.983 (0.956 to 0.993)	0.31 (0.38)
TR (°)	0.73 (1.12)	0.70 (1.12)	0.997 (0.938 to 0.998)	0.86 (0.98)
Tx (mm)	0.02 (0.16)	0.02 (0.18)	0.991 (0.980 to 0.996)	−0.01 (0.11)
Ty (mm)	−0.10 (0.39)	−0.08 (0.36)	0.984 (0.962 to 0.993)	−0.12 (0.42)
Tz (mm)	−0.04 (0.09)	−0.03 (0.07)	0.863 (0.713 to 0.938)	0.03 (0.20)
Rx (°)	−0.16 (0.40)	−0.14 (0.36)	0.972 (0.938 to 0.988)	−0.21 (0.46)
Ry (°)	0.12 (0.50)	0.13 (0.47)	0.978 (0.951 to 0.991)	0.09 (0.74)
Rz (°)	−0.34 (1.11)	−0.34 (1.12)	0.999 (0.999 to 1.000)	−0.27 (0.92)

*Note:* Mean (SD) migrations results in mm/° (*n* = 24) for tibial components in TKA for CT‐RSA (V3MA by 2 observers: OBS1 and OBS2), ICC (95% CI) between OBS1‐OBS2, and RSA migration results (Model‐based RSA).

Abbreviations: mm = millimeter, MTPM = maximum total point motion (mm), Rx, Ry, Rz = rotation about the axes of the migrating coordinate system, SD = standard deviation, ° = degree, TR = total rotation, TT = total translation of center of mass (mm), Tx, Ty, Tz = translations along the axes of the migrating coordinate system.

**FIGURE 4:**
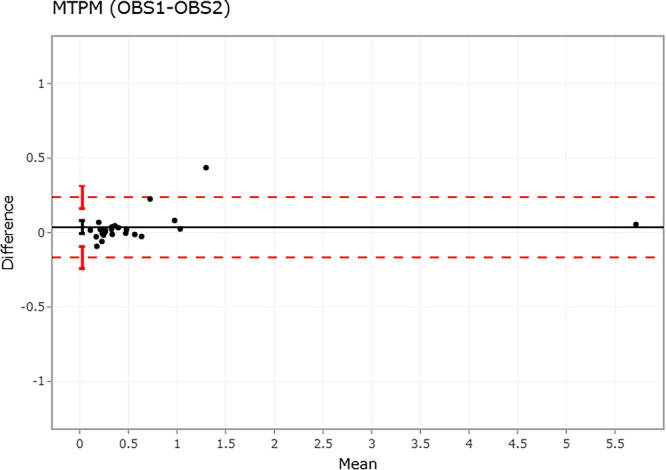
Bland–Altman plot of 24 patients (black dots) comparing observer 1 with observer 2 on MTPM (in mm) using V3MA, showing the mean difference (black solid line) and the limits of agreement (red dashed lines). The vertical lines represent the 95% CI for the mean difference and limits of agreement. MTPM = maximum total point motion. mm = millimeter.

The mean difference (95% CI) in MTPM between observer 1 and observer 2 using V3MA was 0.04 mm (± 0.04), LOA: [−0.17 to 0.24], also indicating no bias (Figure 4). The ICC (95% CI) for MTPM between observer 1 and observer 2 was 0.995 (0.989–0.998), indicating excellent agreement. The lowest ICC was in the anterior‐posterior direction (Tz): 0.863 (0.713 to 0.938). Mean (95% CI) ICC for each migration parameter is shown in Table 1.

Table 2 presents the differences in segmentation between observers, showing a mean absolute difference between threshold values of 78 and 250 hounsfield units for the bone and implant mask, respectively. As a result, the bone masks for observer 2 were slightly larger than for observer 1. These differences in threshold values and bone masks were not significantly different between observers.

**TABLE 2:** CT segmentation details.

	ABS_DIF (*N* = 24)	OBS1 (*N* = 24)	OBS2 (*N* = 24)
**Threshold_Bone (HU)**			
Median, [Min, Max]	78 [0, 300]	430 [230, 770]	360 [220, 520]
**Threshold_Implant (HU)**			
Median, [Min, Max]	250 [0, 920]	2200 [1900, 3100]	2300 [1900, 2800]
**Volume_Bone (CM** ^**3**^ **)**			
Median, [Min, Max]	10 [0.0020, 24]	39 [11, 110]	48 [17, 110]
**Volume_Implant (CM** ^**3**^ **)**			
Median, [Min, Max]	0.76 [0, 3.5]	18 [14, 25]	18 [13, 25]

*Note:* CT‐RSA details of differences in segmentation between observer 1 and observer 2 (OBS1 and OBS2) regarding threshold values (in hounsfield units (HU)) and volume of bone and implant mask.

Abbreviations: ABS_DIF = absolute difference, CM^3^ = cubic centimeters, Max = maximum, Min = minimum, SD = standard deviation.

The median (range) slice thickness of the CT scans was 0.4 mm (0.3–0.5), and the median (range) pixel spacing was 0.5 mm (0.3–0.8); pixel spacing was influenced by the field of view containing a single knee or both knees. The median (range) effective radiation dose was 0.076 mSv (0.021–0.21) for the baseline CT scan and 0.037 mSv (0.015–0.074) for the follow‐up CT scan (CT‐parameters overview in Supplemental Table 1). The median tibia bone length that was used for matching was 7 cm (range: 4–14).

One tibial component was unstable and subsided (Figure 1). For this patient, MTPM was 5.74 mm (CT‐RSA observer1), 5.69 mm (CT‐RSA observer 2) and 5.07 mm (mb‐RSA). The two patients with the largest differences in MTPM between observers (0.44 mm and 0.23 mm) had a CT scan with a pixel size of 0.63 and 0.36 mm, respectively, and tibial bone lengths of 5 cm.

## 4 Discussion

CT‐RSA migration analysis using V3MA was comparable to mb‐RSA analysis using Model‐based RSA software in a clinical follow‐up study between 1 and 5 years for cemented tibial components. Measured migration was low, which was expected since most migration in this group of patients happened in the first 6 months postoperatively, and no group migration between 1 and 2 years was observed [17]. In the current study, the reference timepoint was 1 year postoperative, excluding the interval between direct postoperative and 6 months postoperative, in which most migration normally occurs [22]. No systematic bias was observed for MTPM, translation, or rotation between V3MA and Model‐based RSA, or between two observers both using V3MA. It should be noted that for both V3MA and Model‐based RSA, the default migration coordinate systems were used [15, 17]. Therefore, the origins of the migration coordinate systems are not on the exact same location, and the orientations differ slightly (Figure 1). These differences result in small differences in translations, and rotations, but not in MTPM or TR. Despite this effect, the measured translations and rotations are not different between the migration methods.

The LOA in MTPM between CT‐RSA and mb‐RSA was −0.91 to 0.66 mm and did not exceed ±0.5 mm for translation and ±1.0° for rotations. These LOA are generally in line with the LOA reported by Van Hamersveld et al. (2019) who compared model‐based RSA with marker‐based RSA in the same data set: LOA for MTPM of −0.45 to 0.39 mm and ±0.5 mm and ±0.8° for translations and rotations respectively [23]. Van Hamersveld et al. (2019) concluded that results from model‐ and marker‐based RSA can be pooled, if corrected for differences in the migration coordinate system [23]. The results of the current study indicate that for translations and rotations, and possibly for MTPM as well, this could also apply for migration analysis with CT‐RSA and RSA. An important difference between our study and the study by Van Hamersveld et al. (2019) is that we compared two different analysis methods in two datasets obtained by different imaging modalities (CT scans vs. X‐ray), while Van Hamersveld compared two similar analysis methods in the same data set (X‐ray images only) [23]. This difference could explain the wider LOA in our study.

Engseth et al. (2025) compared CT‐RSA with mb‐RSA to determine tibial migration after TKA in 31 patients with 1‐year follow‐up [24]. They found LOA of –0.34 to 0.98 mm for MTPM between CT‐RSA and mb‐RSA [24]. Contrary to our results, Engseth et al. (2025) found a significant difference of 0.32 mm in MTPM between CT‐RSA measured with CTMA software (Sectra), and mb‐RSA measured with Model‐based RSA software [24]. They also reported a significant difference between CT‐RSA and mb‐RSA for medial translation (0.10 mm vs. −0.01 mm) and transversal rotation (tilt, −0.04° vs. −0.31°) [24]. Our study does not indicate a difference between CT‐ and mb‐RSA on any of the migration parameters. In agreement with the study by Engseth et al. (2025), we found lower MTPM values for CT‐RSA compared to mb‐RSA, although in our study, this difference was not significant. As MTPM is an absolute value, its mean value is influenced by measurement noise [25]. Therefore, lower mean MTPM values in a clinical study are indicative for lower noise levels and less migration. The observed difference in MTPM in Engseth et al. (2025), but not in the current study, could be related either to the data set, or to the CT‐RSA methods used. CTMA uses surface registration [26], whereas V3MA uses voxel intensities and thereby volume registration [15]. Currently, no direct comparison between different CT‐RSA methods is available.

To assess interobserver agreement of V3MA for tibial implant migration, we compared MTPM measurements between two independent observers. The interobserver agreement was excellent with an ICC of 0.995 for MTPM, indicating that the two observers calculated consistent results. Combined with the Bland–Altman analysis that showed no bias between the observers, this indicates that both users of V3MA obtained almost identical results. Few studies reporting on interobserver agreement between CT‐RSA and RSA have been published and none specifically on tibial implants in a clinical study. In a porcine model validating precision of CT‐RSA with RSA of a tibial implant using repeated measures, moderate to good ICC for all migration parameters combined was reported: 0.77 (CI 0.72–0.82). However, ICC on individual translations, rotations, and TT varied [27]. Sandberg et al. (2020) determined interobserver agreement for a femoral stem in a clinical study, which showed excellent agreement: ICC's > 0.87 for CTMA [28]. In the current study, the ICC of MTPM was 0.995, and for anterior‐posterior translation, the ICC was 0.863. All others migration parameters ICC were above 0.95 (Table 1).

Though the studies from Engseth, Sandberg, and the current study use different study designs and different CT‐RSA software, the interobserver agreements of CT‐RSA methodologies appear to be good.

MTPM is considered an important metric to assess stability and safety of tibial implants [29], and relevant for prognostic use [11]. The observers in the current study were both trained by the developer of V3MA software (B.K.) in segmentation and V3MA usage, and the results were evaluated with the developer. The excellent ICC of the current study on MTPM shows that CT‐RSA results determined with V3MA are reliable results and seem to be independent of V3MA analyst.

De Laat et al. (2024) state that a knee CT scan has an effective dose (ED) of 0.16 mSv, whereas paired RSA radiographs have an ED of 0.003 mSv [15]. The median ED in the current study was 0.076 mSv at 1 year (baseline) and 0.037 mSv at 5 year (follow‐up), sufficiently low to acquire several CT scans in clinical research to increase general knowledge [30].

The two patients for whom the difference in MTPM between observers was largest had proximal tibial bone lengths of approximately 5 cm. The proximal tibial bone is used to align the baseline and follow‐up CT scans. Therefore, in theory, the length of the bone that is visible in both scans and therefore available for registration, could affect registration accuracy, which could result in larger differences between observers or methods. Sensitivity analysis (data not shown) of interobserver agreement and of CT‐RSA and mb‐RSA differences with short and longer tibial bone lengths did not show different results. However, we do recommend including at least 7 cm of tibial bone in the CT scans to ensure a good registration is achieved. This recommendation is in line with the practical guidelines on CT‐based RSA, where 5 cm below the implant as recommended [31].

### 4.1 Strengths and Limitations

The current study was designed to evaluate tibial migration in a cohort of TKA patients, measured with CT‐RSA and mb‐RSA. Strong points of this study are that patients were included from a RCT and were invited for a medium‐term follow‐up at 5 years. At present, this is the longest follow‐up study on tibial implant migration measured with CT‐RSA. The 4‐year interval between baseline and follow‐up in this study enabled the capture of possible changes in bone morphology over time, including osteophyte formation, remodeling, stress shielding, and implant‐related osteoporosis. All factors could change bone shape and density, and could thus affect image registration and CT‐RSA. Nevertheless, the absence of a systematic bias between CT‐RSA and mb‐RSA, in combination with the observed LOA comparable to those reported by Van Hamersveld et al. [23], indicate that V3MA is robust despite the extended follow‐up.

A further strong point is the use of standard clinical CT protocols, resulting in variations in image resolution between both patients and time points. This reflects clinical practice and enhances the generalizability of findings.

Furthermore, 2 observers conducted the analysis independently using different threshold values for segmentation of the baseline CT scans. In V3MA, segmentation serves solely to define the image mask for selecting voxel intensities (gray values) used for registration. Despite variations in thresholding, both observers produced near‐identical results indicating user independency of V3MA.

Nevertheless, some limitations exist.

The current study is not a clinical migration study and for medical ethical considerations regarding radiation dose, clinical precision of the migration methods was not determined, such as is recommended for migration studies [18]. We measured comparable values for TT, TR, and MTPM for V3MA compared to Model‐based RSA. These absolute measures are known to be influenced by measurement noise [18, 32], indicating that the precision of CT‐RSA analysis is similar to RSA analysis precision. In addition, a previous study by Hext et al. showed comparable clinical precision between V3MA and Model‐based RSA [33]. They reported tibial MTPM precision (1.95*SD) of 0.14 and 0.17 mm for V3MA and Model‐based RSA, respectively. For total translation, precision values were 0.06 mm compared to 0.06 mm, and for total rotation, precisions were 0.12° and 0.27°, respectively [33]. In addition, a recent systematic review on accuracy and precision of CT‐RSA reported pooled precision values of 3 in‐vitro studies on tibial TKA prostheses [15, 27, 34], showing that CT‐RSA has in general similar precision to RSA: translations of 0.04 mm (95% CI 0.00 to 0.08, 95% Prediction Interval (PI) −0.01 to 0.09) and rotations of 0.07° (95% CI 0.00 to 0.015, 95% PI −0.01 to 0.15) [16].

This study used the 1‐year follow‐up as a baseline for migration calculation at 5 year postoperative. Bone remodeling is likely to occur to some extent after surgery. Ideally, RSA images and CT scans would have been obtained shortly after surgery to capture as much bone remodeling as possible, but at the time of designing the original clinical study, there was no need for a direct postoperative CT scan.

In RSA, the mean error of rigid body fitting and condition number are indicative of the stability and 3D‐distribution of the bone‐markers [18]. For CT‐RSA, such quality metrics are not yet available.

Another limitation of using a 1‐year follow‐up as a baseline is that the majority of migration of TKA components is not captured, as this usually occurs in the initial 6 months postoperatively [22], and the presented migration in this study cannot be compared to migration studies of tibial implants. This study showed low migration values at the 5‐year follow‐up, with only one patient with > 5 mm MTPM. All other patients had MTPM values below 1.52 mm. The absence of larger migrations could mask a possible systematic error in clinical CT‐RSA measurements when evaluating the Bland–Altman plot. The previous in vitro accuracy and precision study performed on tibial implants [15] and the clinical study by Engseth et al. [24] do not give an indication for a systematic error in CT‐RSA measurements.

No sample size calculation was performed to determine the amount of patients to be included in the study. We tried to include as many patients as possible from a fixed cohort of 54 patients.

Furthermore, V3MA was only tested in one type of CT‐scanner using a standard clinical CT protocol with a CT resolution of approximately 1.0 lp/mm. The slice thickness (median 0.4 mm, range 0.3–0.5) and pixel size (median 0.5 mm, range 0.3–0.8) are contributing factors for the spatial resolution of the CT scan, which could influence accuracy of migration measurements, as suggested before [14] In this study, only CT scans with a slice thickness < 1.0 mm were included in the analysis. Institutional clinical CT scans have been observed with larger slice thicknesses. Therefore, the presented results of CT‐RSA cannot be generalized to any clinical CT scan (different CT‐scanners, larger slice thicknesses, etc.). However, the results from the precision study by Engseth et al. [27] and the systematic review by Vusse et al. [16] suggest that CT‐RSA is accurate and precise regardless of CT‐scanner, joint, and CT‐RSA method used. Regarding slice thickness, it is advised to clearly specify the settings for the CT scanner to have a slice thickness below < 1.0 mm [18], but preferably < 0.6 mm as recommended in the practical guidelines for CT‐RSA measurements [31].

## 5 Conclusion

In conclusion, migration data of tibial TKA components is comparable between V3MA and Model‐based RSA analysis and interobserver agreement for V3MA is excellent. Therefore, V3MA can be used for medium‐term clinical follow‐up studies measuring tibial TKA component migration with similar accuracy to Model‐ based RSA. However, further longer‐term clinical studies with V3MA in different joint implants are required before general use of V3MA in clinical studies.

## Author Contributions

Nienke N. de Laat performed V3MA analysis, statistics, and drafted the manuscript. Jessie E. Robertson performed V3MA analysis as the second observer. Lennard A. Koster performed RSA analysis and drafted the manuscript. Bart L. Kaptein supervised the work and drafted the manuscript. Rob G.H.H. Nelissen and Bart L. Kaptein conceptualized the project. All authors read and approved the final manuscript.

## Supporting information

Resubmission Clinical Validation Inter‐Observer Agreement V3MA‐DeLaat Supplemental Figures Tables 20250729.
