# Supplementary material for: Clinical Validation and Excellent Interobserver Agreement of Volumetric Matching Micromotion Analysis (V3MA) in Total Knee Arthroplasty
Source: J Orthop Res. 2025 Aug 24;44(1):e70049. doi: 10.1002/jor.70049 (PMC12701625; doi:10.1002/jor.70049)
Supplement: Supplementary file 1 — Resubmission Clinical Validation Inter‐Observer Agreement V3MA‐DeLaat Supplemental Figures Tables 20250729. [file JOR-44-0-s001.docx]

**Supplemental figure 1:**

**Graphical overview of segmentation and registration.**Figures A – D show an overview of the segmentation and registration process for migration analysis in V3MA. Each image is at the same anterior-posterior plane.

A= Baseline CT scan;

B = Baseline CT scan, including bone masks (Blue: femur and tibia masks; red: tibial implant mask); Note that the blue masks contain noise due to metal artefacts.

C = Baseline CT scan with the masks used for registration (green: tibial bone mask; red: tibial implant mask). Note that the tibial bone mask used for registration does not contain the most proximal part of the tibia bone mask from B, which contains noise due to metal artefacts.

D = Checkerboard view used for visual inspection of registration of baseline and follow-up CT scans.


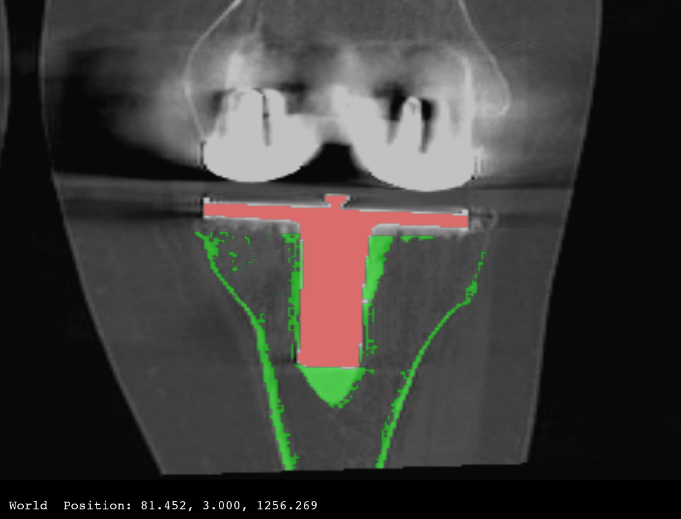


**C**


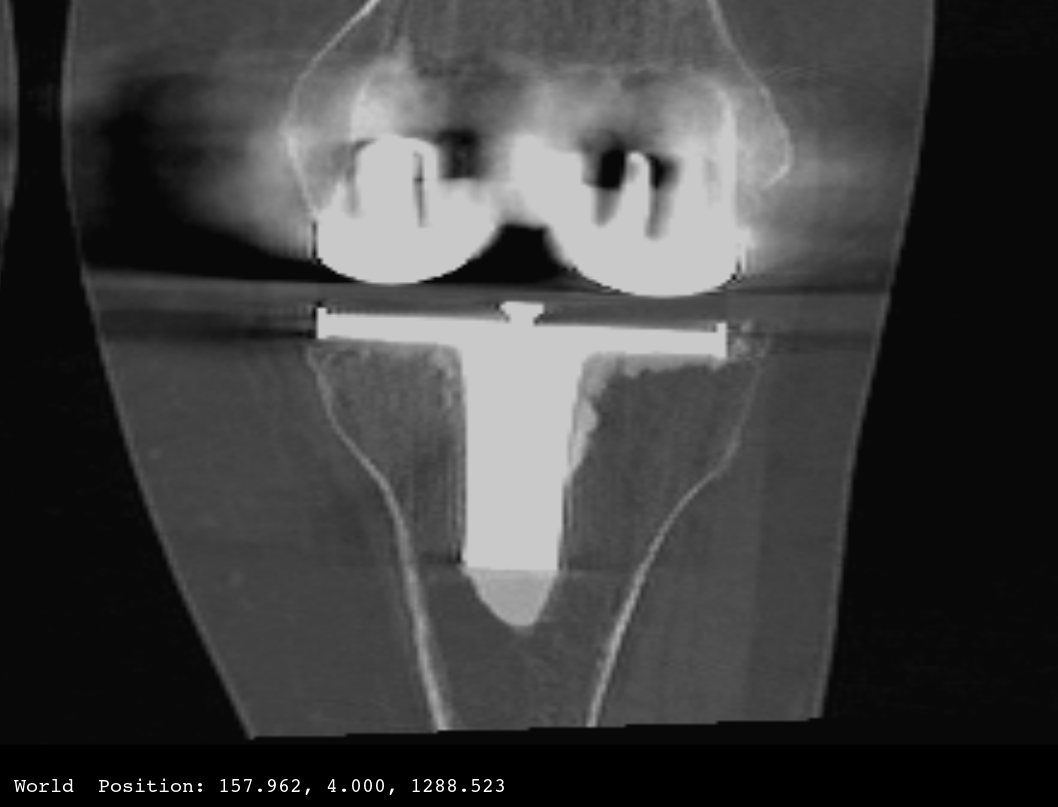


**A**


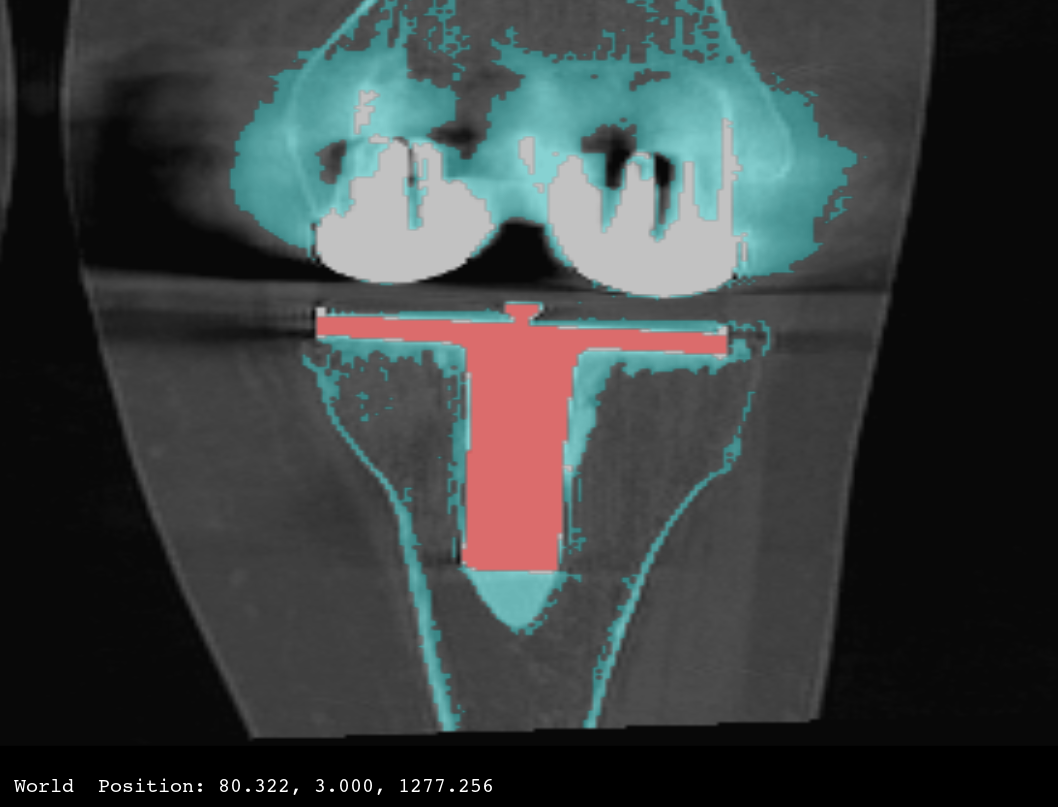


**B**


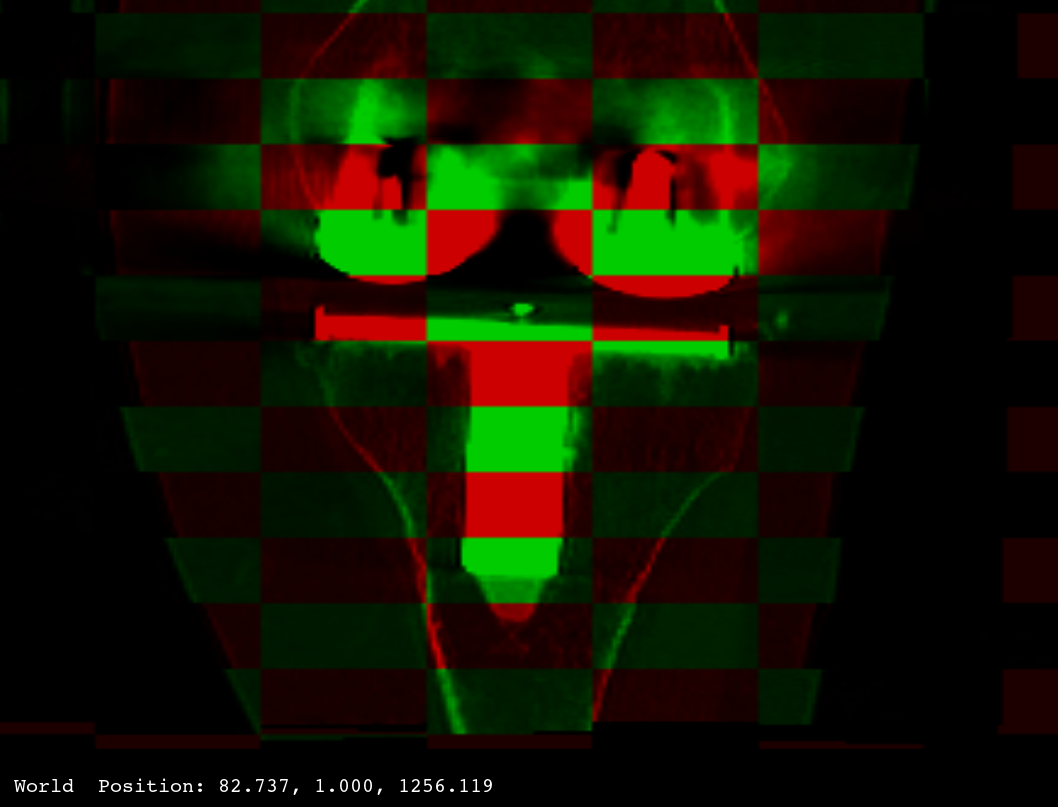


**D**

**Supplemental figure 2:**

**Bland-Altman plots** of 24 patients (black dots) comparing V3MA (OBS 1) with Model-based RSA on Tx, Tz and TT (in mm) and Ry, Rz and TR (in °) showing the mean difference (black solid line) and the limits of agreement (red dashed lines). The vertical lines represent the 95% CI for the mean difference and limits of agreement. Tx, Tz and TT = medial-lateral translation, anterior-posterior translation and total translation; Ry, Rz and TR = internal-external rotation, varus-valgus rotation and total rotation; mm = millimeter; ° = degree.


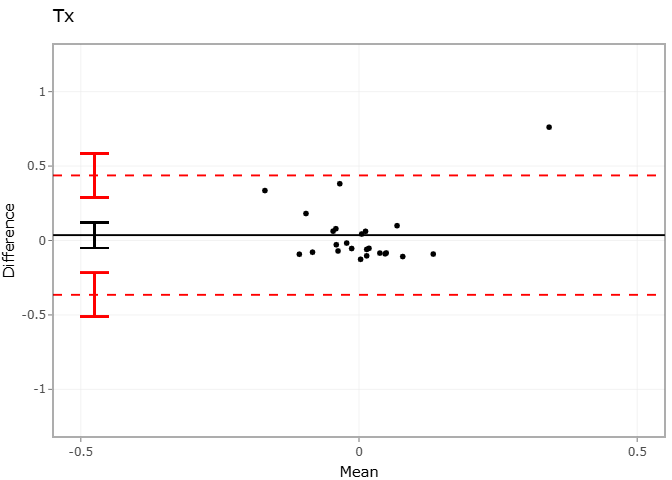

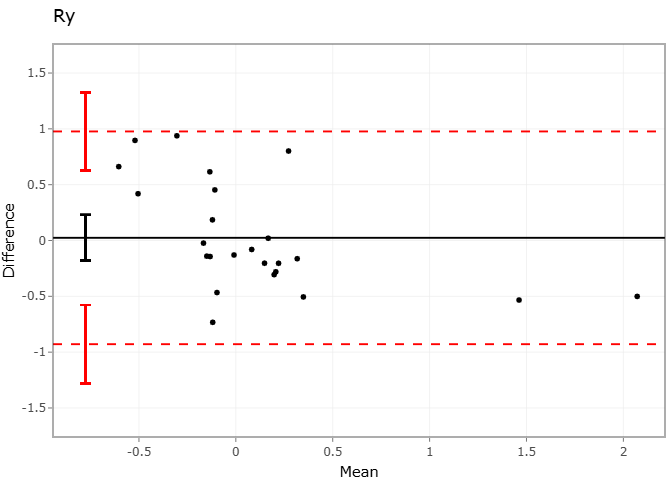

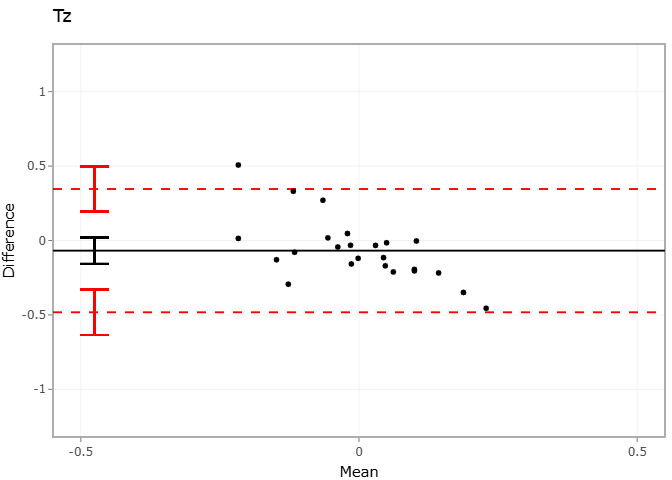

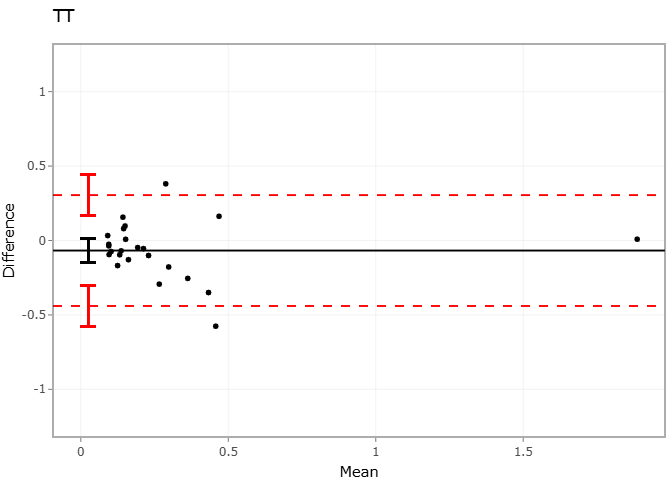

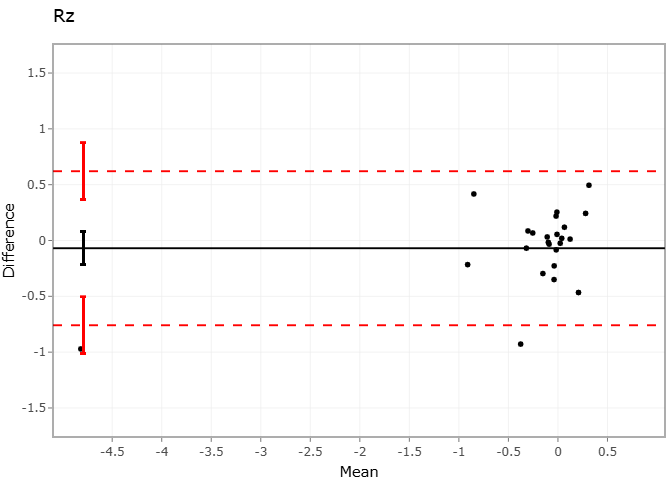

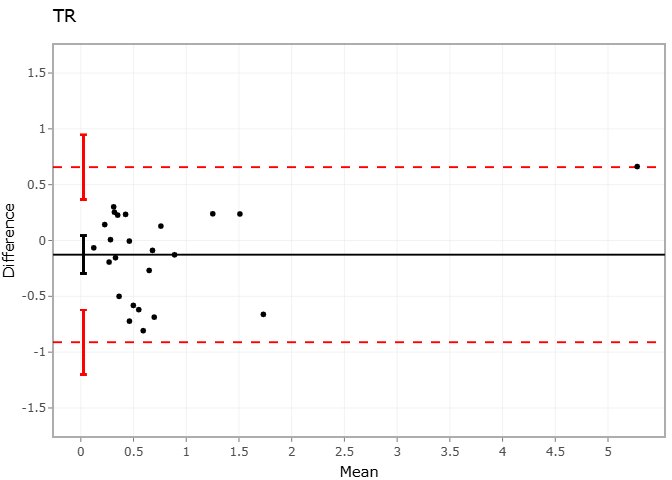


**Supplemental Table 1:**

**Overview of CT parameters.**
Median (range) CT-parameters of CT scans at one year (YR1) and five years (YR5) after primary total knee arthroplasty. All scans have a matrix size 512x512 and convolution kernel FC30 (Toshiba convolution kernel used in image reconstruction).
FU = follow up, CT = Computed Tomography, kVp = kilo volt, mA = milli-ampere, mm = millimeter, mGy = milli-gray.

| **FU** |  | **Slice thickness (mm)** | **pixel spacing (mm)*** | **CT tube voltage (kVp)** | **CT tube current (mA)** | **Data collection diameter (mm)** | **Reconstruction diameter (mm)** | **Dose (mGy)** |
| --- | --- | --- | --- | --- | --- | --- | --- | --- |
| **YR1** | Median | 0.4 | 0.43 | 135 | 100 | 400 | 221 | 0.083 |
|  | Range | 0.3 - 0.5 | 0.27 - 0.78 | 120 - 135 | 30 - 250 | 320 - 500 | 140 - 400.39 | 0.039 - 0.239 |
| **YR5** | Median | 0.4 | 0.405 | 120 | 110 | 320 | 203.75 | 0.041 |
|  | Range | 0.3 - 0.5 | 0.30 - 0.63 | 120 - 135 | 30 - 150 | 240 - 500 | 152.5 - 321.88 | 0.024 - 0.079 |

*Pixel spacing (voxel size) is equal in both directions (square) and equals pixel size assuming the space between pixels is zero.
